# The Structure and Function of the Gram-Positive Bacterial RNA Degradosome

**DOI:** 10.3389/fmicb.2017.00154

**Published:** 2017-02-03

**Authors:** Kyu Hong Cho

**Affiliations:** Department of Biology, Indiana State UniversityTerre Haute, IN, USA

**Keywords:** Gram-positive RNA degradosome, *B. subtilis*, *S. aureus*, *S. pyogenes*

## Abstract

The RNA degradosome is a highly structured protein complex responsible for bulk RNA decay in bacteria. The main components of the complex, ribonucleases, an RNA helicase, and glycolytic enzymes are well-conserved in bacteria. Some components of the degradosome are essential for growth and the disruption of degradosome formation causes slower growth, indicating that this complex is required for proper cellular function. The study of the *Escherichia coli* degradosome has been performed extensively for the last several decades and has revealed detailed information on its structure and function. On the contrary, the Gram-positive bacterial degradosome, which contains ribonucleases different from the *E. coli* one, has been studied only recently. Studies on the Gram-positive degradosome revealed that its major component RNase Y was necessary for the full virulence of medically important Gram-positive bacterial pathogens, suggesting that it could be a target of antimicrobial therapy. This review describes the structures and function of Gram-positive bacterial RNA degradosomes, especially those of a Gram-positive model organism *Bacillus subtilis*, and two important Gram-positive pathogens, *Staphylococcus aureus* and *Streptococcus pyogenes*.

## Introduction

Bacteria change the expression of genes to adapt to the environmental conditions, and this leads to the fluctuation of transcript abundance according to growth status or environment conditions. The cellular mRNA level is decided by the synthesis and degradation rates of each mRNA. Bacteria have been known to have very rapid mRNA turnover rates with an average half-life of several minutes (Hambraeus et al., 2003; Bernstein et al., 2004; Kristoffersen et al., 2012; Marincola et al., 2012). This rapid mRNA turnover rate confers speedy adjustment to environmental changes, so is a key to regulate gene expression in response to different environmental conditions.

RNA degradation is performed by RNases. Some RNases are specific for certain RNA degradation and processing events, and others have broad substrate specificity that is involved in bulk RNA degradation. Even though most RNA synthesis machinery between Gram-positive and Gram-negative bacteria is similar, the machinery for bulk RNA degradation between them is quite different. For example, the Gram-positive organism *Bacillus subtilis* does not contain RNase E, RNase II, poly(A) polymerase, and oligoribonuclease, but contains some novel enzymes that do not exist in *Escherichia coli* (Commichau et al., 2009; Lehnik-Habrink et al., 2012). Generally, the major RNases involved in bulk RNA turnover form protein complexes for efficient and regulated degradation. These protein complexes in bacteria are called the RNA degradosome. The RNA degradosome is believed to be the major cellular structure to degrade most RNAs in bacteria, thereby influencing the average half-life of bulk RNA messages (Górna et al., 2012). The bacterial RNA degradosomes often include endoribonucleases, exoribonucleases, ATP dependent-RNA helicases, and glycolytic enzymes. Most Gram-negative bacteria including α- and γ-proteobacteria use the membrane-bound endoribonuclease RNase E as the scaffold for the complex. However, most Gram-positive bacteria do not have RNase E homologs in their genomes. Instead, they use the membrane-anchored endoribonuclease RNase Y (aka CvfA) as a major scaffold for the RNA degradosome complex (Table 1). The *E. coli* RNA degradosome has been intensively studied for decades, so the structures and function of the complex and the individual members have been well-demonstrated *in vivo* and *in vitro*. However, the RNA degradosome of Gram-positive bacteria had not been studied until recent discovery of RNase Y, so fewer studies have been done compared to the Gram-negative RNA degradosome.

**Table 1:** **Comparison between the RNA degradosomes of ***E. coli*** and ***B. subtilis*****.

**Microorganism**	***E. coli***	***B. subtilis***
Phylogenetic description	A Gram-negative gamma-Proteobacterium	A Gram-positive Firmicute
Major components	RNase E, PNPase, RhlB, Enolase	RNase Y, RNase J1 and J2, PNPase, CshA, Enolase, Phosphofructokinase (PFK)
Enzymes with endoribonuclease activity	RNase E	RNase Y, RNase J1 and J2
Enzymes with 5′ → 3′ exoribonuclease activity	None	RNase J1 and J2
Enzymes with 3′ → 5′ exoribonuclease activity	PNPase	PNPase
Enzymes with ATP dependent RNA helicase activity	RhlB	CshA
Glycolytic enzymes	Enolase	Enolase and PFK
Scaffold protein for the degradosome assembly	C-terminal half of RNase E	RNase Y
Essentiality of the scaffold protein for cell growth	N-terminal half endoribonuclease domain of RNase E: essential	RNase Y: not essential
	C-terminal half scaffolding domain of RNase E: not essential	
Location of the degradosome	Attached to the membrane through the membrane targeting sequence in the C-terminal half of RNase E	Anchored to the membrane through the N-terminal transmembrane domain of RNase Y

Recent studies revealed that the RNA degradosome (or at least RNase Y) plays a crucial role in maintaining the virulence of Gram-positive pathogens. Here general features and influence to virulence of the Gram-positive RNA degradosomes of *B. subtilis, Saphylococcus aureus*, and *Streptococcus pyogenes* are reviewed.

## Structure and function of the gram-positive RNA degradosome

### *B. subtilis* RNA degradosome

To describe the Gram-positive RNA degradosome better, understanding the well-studied *E. coli* RNA degradosome first would be helpful. The *E. coli* RNA degradosome complex includes the endoribonuclease RNase E, the exoribonuclease polynucleotide phosphorylase (PNPase), the DEAD-box RNA helicase RhlB, and the glycolytic enzyme enolase (Table 1). The degradosome is the major player involved in degrading most RNA transcripts and maintains the typical half-lives of RNA transcripts (Carpousis, 2007). The endoribonuclease RNase E is responsible for the decay of bulk mRNA. Mutations in RNase E influence the half-lives of ~60% of *E. coli* mRNA (Bernstein et al., 2004; Stead et al., 2011). Also, it acts as a scaffold, recruiting the other proteins and organizing the structure of the RNA degradosome (Górna et al., 2012).

RNase E has two major domains: N-terminal ribonuclease domain (NTD) and C-terminal scaffolding domain (CTD; Górna et al., 2012). The N-terminal half of RNase E has endoribonuclease activity, which is the major activity initiating the decay of bulk mRNA in the RNA degradosome and essential for the growth of *E. coli*. The C-terminal half of RNase E is intrinsically unstructured except six microdomains mostly involved in interactions with other components in the degradosome. The secondary structure of this half is not much predicted by *in silico* analysis, indicating that this part is unstructured and does not form a stable globular structure (Gunasekaran et al., 2003). Even though most sequences of the C-terminal half are not much conserved among RNase E orthologs, some parts are more conserved than others. These conserved microdomains range from 20 to 70 residues in size and are binding sites for other protein components in the RNA degradosome, RNA substrates, and the cytoplasmic membrane (Vanzo et al., 1998; Callaghan et al., 2004; Chandran et al., 2007; Khemici et al., 2008). The first microdomain in the C-terminal half, the membrane targeting sequence (MTS), forms an amphipathic helix that interacts with the membrane.

*B. subtilis* is a spore-forming soil bacterium whose RNA degradosome was discovered first among Gram-positive bacteria (Commichau et al., 2009). *B. subtilis*, a facultative anaerobe, has been studied intensively, especially in the area of chromosome replication and sporulation, and considered as a Gram-positive model organism. The *B. subtilis* RNA degradosome contains similar or the same components as the *E. coli* RNA degradosome such as a DEAD-box helicase, the glycolytic enzyme enolase, and the 3′–5′ exoribonuclease PNPase. Like in *E. coli*, these components are also found in the cytoplasm (Cascante-Estepa et al., 2016), suggesting that these proteins fulfill independent functions in the cytoplasm and only a fraction of them interact with the degradosome. However, the *B. subtilis* degradosome does not have a homolog of RNase E, the scaffold protein in *E. coli*. Instead, the degradosome contains new endoribonucleases that are not found in the *E. coli* degradosome. These novel enzymes are RNase Y, RNase J1, and RNase J2. Among these three endoribonucleases, RNase Y and RNase J1 are involved in the degradation of bulk mRNA (Even et al., 2005; Shahbabian et al., 2009; Durand et al., 2012).

When RNase Y is depleted, the average half-life of bulk mRNA increases more than 2-fold (Shahbabian et al., 2009). Also, RNase Y influences the steady-state level of about 25% of transcripts (Lehnik-Habrink et al., 2011a; Durand et al., 2012), indicating that RNase Y plays a crucial role in global mRNA turnover. Like RNase E, RNase Y is an endoribonuclease with a preference for 5′ monophosphorylated ends (Shahbabian et al., 2009). In addition, it has 5′ end independent endoribonuclease activity against certain transcripts (Yao et al., 2011). RNase Y also has the ability to interact with all other enzymes in the degradosome except RNase J2 (Commichau et al., 2009). At first, RNase Y was considered essential for growth. However, a recent study shows that it is not essential but the null mutant pays a very high fitness cost for growth (Figaro et al., 2013).

**Figure 1:**
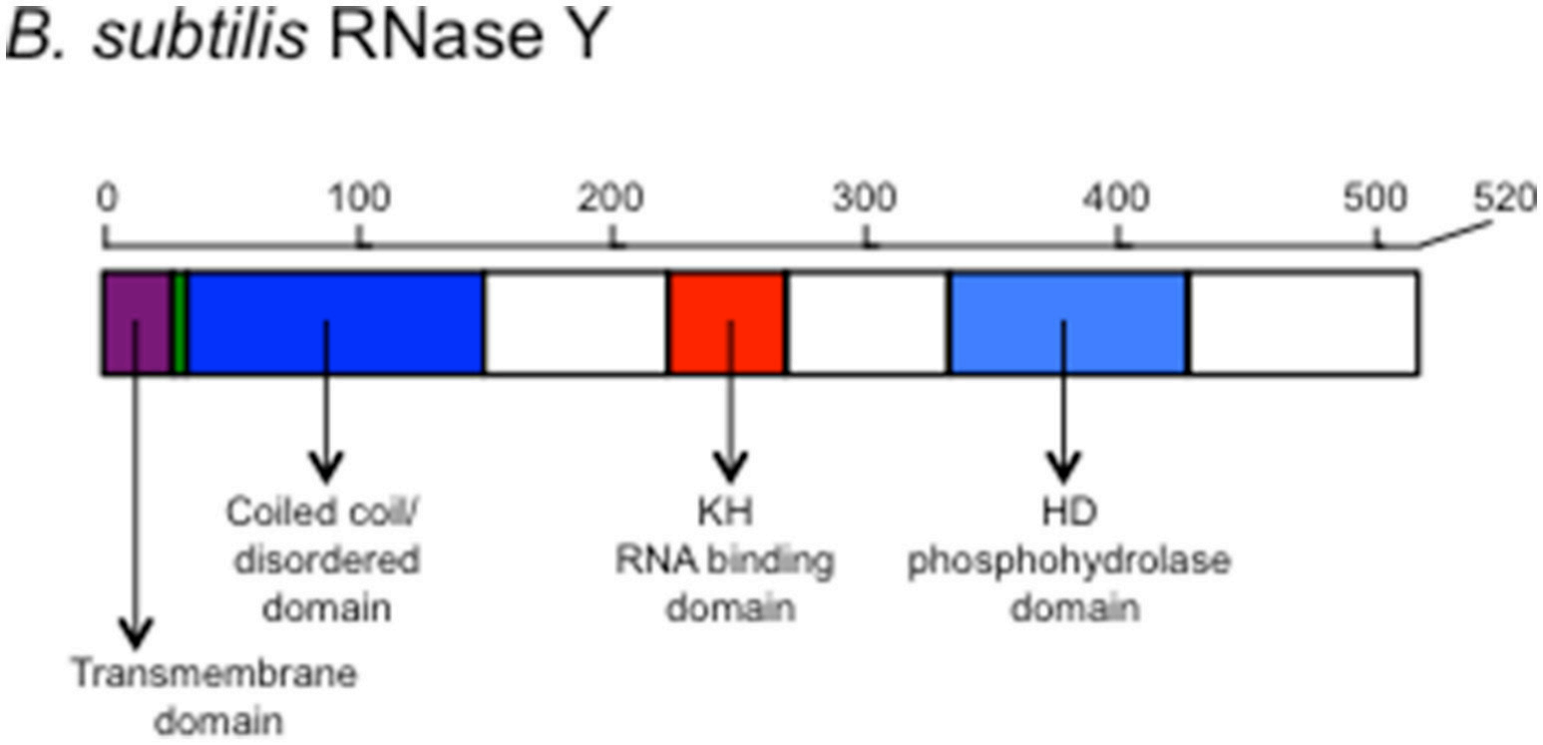
**The schematic representation of the scaffold protein of the Gram-positive bacterial RNA degradosome, RNase Y**. Structural and functional domains are shown. The transmembrane domain is used for anchoring on the membrane. The coiled-coil/disordered domain is speculated for a dimer or multimer formation of RNase Y and for a place where other members of the degradosome bind. The KH domain contains an RNA binding motif determining the RNA substrate specificity and the HD domain has a ribonuclease activity.

**Figure 2:**
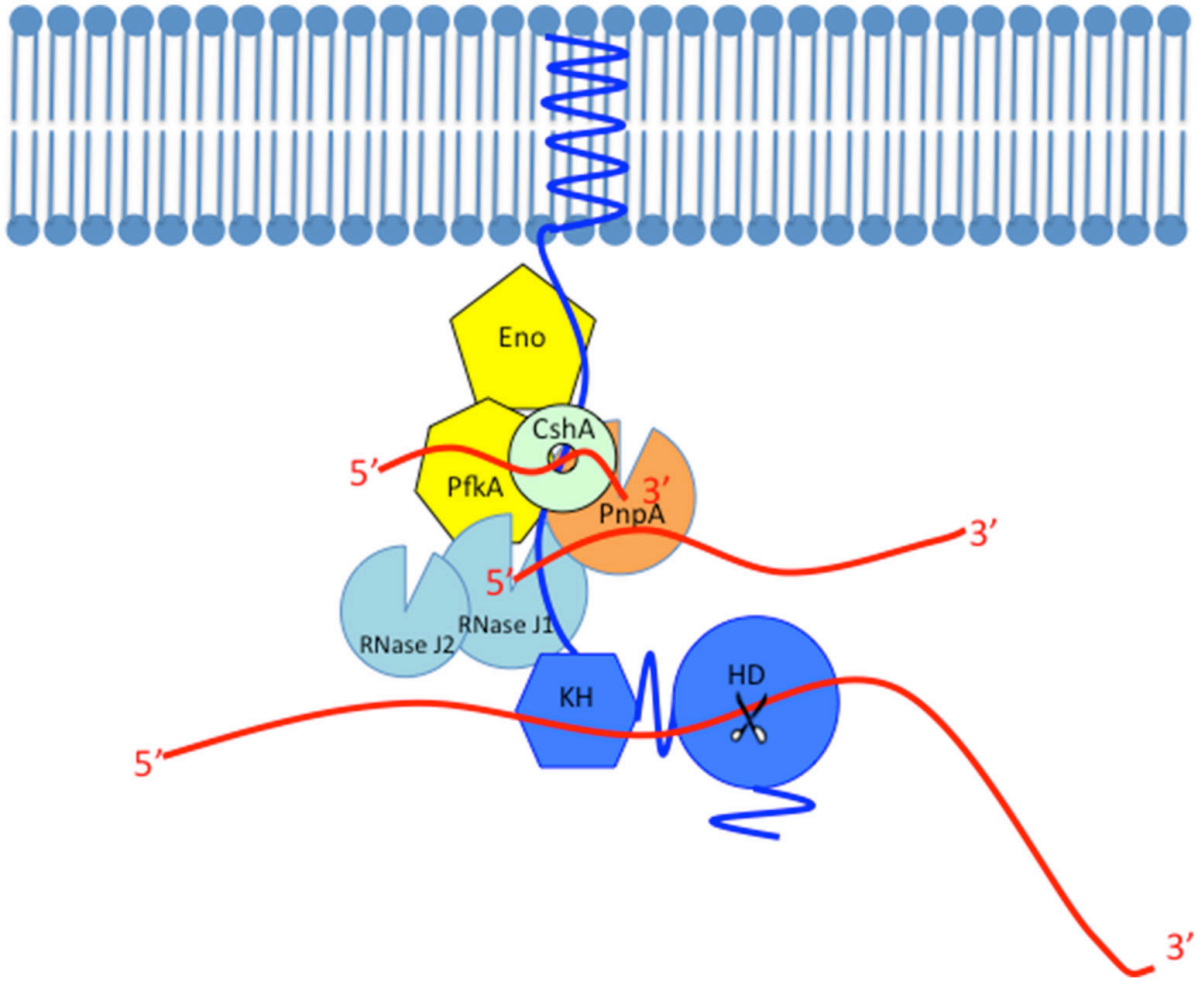
**Model of the Gram-positive RNA degradosome**. The scaffold protein of the RNA degradosome RNase Y (shown in blue) is anchored to the membrane through its transmembrane domain, interacts with other members probably through the coiled-coil domain, binds with its RNA substrates through the KH domain and cleaves the substrate through the HD domain whose endoribonuclease activity is shown as scissors. The single-stranded RNAs are shown as red lines. RNase Y interacts with most members in the RNA degradosome such as the glycolytic enzymes (shown in yellow), enolase (Eno), and phosphofructokinase (PfkA), the DEAD-box RNA helicase CshA (shown in light green or mint), the 3′–5′ phosphorolytic exoribonuclease PNPase (PnpA, shown in orange), and the exo/endo-ribonuclease RNase J1 (shown in light blue). This figure only shows the robust 5′–3′ exoribonuclease activity of RNase J1. The RNase J1 paralog with lower 5′–3′ exoribonuclease activity, RNase J2 is also shown in light blue.

*B. subtilis* RNase Y has four major domains: N-terminal transmembrane domain, a coiled-coil domain, an RNA-binding KH domain, and a catalytic HD domain, in that order (Figure 1). The interaction with the membrane occurs through the N-terminal transmembrane domain and is important for the function of the RNA degradosome (Figure 2; Lehnik-Habrink et al., 2011b). The following domain is a coiled-coil domain, which seems to be intrinsically disordered in solution and important for self-oligomerization. Similar to the C-terminal half of RNase E, the disordered region might play an important role in protein-protein interactions. Structured microdomains in disordered regions can be relatively free from the structural and thermodynamic constraints of protein folding (Lehnik-Habrink et al., 2011b). The disordered region in RNase Y has not yet been studied in detail. The KH domain in RNase Y is an RNA binding domain that contains ~70 amino acids. KH domains can bind single stranded RNA or DNA and are involved in different biological processes such as transcriptional controls, translational controls, or splicing (Valverde et al., 2008). KH domains are classified into two types: Type I KH domains are found in multiple copies in eukaryotic proteins and Type II domains are found in generally a single copy in prokaryotic proteins. Interestingly, the KH domain in RNase Y is Type I with a single copy, so the KH domain in RNase Y is uncommon in bacteria. KH domains usually have broad substrate specificity. The typical binding surface of KH domains can accommodate only four consecutive RNA bases. Since the depletion of RNase Y increases the half-life of bulk mRNA significantly, RNase Y appears to have broad substrate specificity (Shahbabian et al., 2009).

In addition, RNase Y is often used for specific cellular events in *B. subtilis*. RNase Y cleaves between the *cggR* and *gapA* transcripts in the *gapA* operon that encodes six genes in the glycolytic pathway (*cggR, gapA, pgk, tpiA, pgm*, and *eno*), and this cleavage separates the expression level between the regulator CggR and the glycolysis structural genes by conferring differential stability to the transcripts (Commichau et al., 2009). Also, it initiates the degradation of all 11 SAM-dependent riboswitches only when the transcripts are prematurely terminated by binding to S-adenosylmethionine (SAM; Shahbabian et al., 2009).

The other ribonucleases found only in the *B. subtilis* degradosome, but not in the *E. coli* one, are RNase J1 and J2 (Figure 2). When RNase J1 was discovered, it was thought to be the functional homolog of RNase E because RNase J1 is a new class enzyme that can initiate RNA degradation. RNase J1 possesses two enzymatic activities: 5′–3′ exoribonuclease activity and 5′ end independent endoribonuclease activity (Even et al., 2005). Recent biochemical and structural studies show that the major enzymatic activity of RNase J1 is the 5′–3′ exoribonuclease activity (Condon, 2010; Newman et al., 2011). It has robust 5′–3′ exoribonuclease activity *in vitro* and the exoribonuclease activity is required to process 16S rRNA from the 5′ end *in vivo* (Mathy et al., 2007). However, the endoribonuclease activity was detected only *in vitro* with high protein concentration, so the endoribonuclease activity of RNaseJ1 is highly controversial (Condon, 2010). RNase J1 and J2 were shown to affect gene transcription on a global scale but not as much as RNase E (Mäder et al., 2008). Recently, RNase Y has been proposed to be the functional homolog of RNase E because of (i) its localization on the membrane, (ii) its interaction with other protein members in the RNA degradosome, (iii) its endoribonuclease activity with the same substrate preference as RNase E that prefers 5′ monophosphorylated RNA, and (iv) its significant influence on average half-life of bulk mRNA.

Before RNase J1 and J2 were discovered, it had long been believed that, unlike eukaryotes, bacteria did not possess enzymes with 5′–3′ exoribonuclease activity. For example, *E. coli* has three 3′–5′ exoribonucleases: PNPase, RNase R, and RNase II, but no 5′–3′ exoribonucleases. However, the discovery of RNase J1 changed this notion because many Gram-positive bacteria have RNase J1 orthologs. The substrates for the RNase J1 exoribonuclease activity are 5′ non- or monophosphorylated RNA (Mathy et al., 2007). Therefore, the substrates can be generated by the action of endoribonucleases that produce monophosphorylated 5′ end or the pyrophosphohydrolase RppH that removes diphosphate from 5′ triphosphate of nascent transcripts (Richards et al., 2011).

In *B. subtilis*, both RNase J1 and RNase J2 are not essential for growth but RNase J1 null mutants display more severe growth defect than RNase J2 null mutants (Figaro et al., 2013). The exoribonuclease activity of RNase J1 is 25–100 fold higher than that of RNase J2. (Mathy et al., 2010). RNase J1 and J2 form a heterotetramer (Mathy et al., 2010), and RNase J2 attaches to the degradosome only through the interaction with RNase J1 (Commichau et al., 2009). Many Gram-positive bacteria have both RNase J1 and J2, implying that these two proteins have their own unique roles *in vivo*, even though some of their roles could be overlapped.

PNPase, which is a 3′–5′ phosphorolytic exoribonuclease, is also a member of the RNA degradosome. *B. subtilis* has four 3′–5′ exoribonucleases: PNPase, RNase R, RNase PH, and YhaM. Among these, PNPase is the major exoribonuclease in the RNA degradosome. Unlike the other 3′–5′ exoribonucleases that use a hydrolytic mechanism, PNPase uses a phosphorolytic mechanism to degrade RNA, so PNPase activates an inorganic phosphate to attack the 3′ phosphodiester bond in RNA and produces a single diphosphate nucleoside as the result. PNPase is not essential for growth but appears to be crucial for cellular and metabolic activities. A *pnpA* mutant is cold sensitive, forms filaments, and loses competence (Luttinger et al., 1996; Wang and Bechhofer, 1996). When endoribonucleases cleave internal regions of transcripts, PNPase binds the endonucleolytically generated 3′ sites and degrades the transcripts (Figure 2; Deikus and Bechhofer, 2007). PNPase is processive, so it continues to cleave along the same RNA substrate. However, since the phosphorolytic mechanism generates little free energy change in RNA degradation, PNPase alone often cannot degrade structured RNA with extensive stem and loop structures. Hence, RNA helicases are required for the complete digestion of structured RNA (Coburn et al., 1999; Khemici and Carpousis, 2004).

Another component of the RNA degradosome is a DEAD-box RNA helicase. DEAD-box RNA helicases, which have D-E-A-D amino acid residues within RecA-like domains, act locally to unwind only a few base pairs of duplex RNA at the expense of ATP hydrolysis. Four ATP-dependent DEAD box helicases exist in *B. subtilis*. Among these, CshA can interact with RNase Y, PNPase, enolase, and phosphofructokinase (PFK). These interactions suggest that CshA is a member of the degradosome and the functional equivalent of RhlB that is the major DEAD-box helicase found in the *E. coli* degradosome (Lehnik-Habrink et al., 2010).

Among the major components of the RNA degradosome, only glycolytic enzymes are not directly involved in the degradation of RNA. In the *E. coli* degradosome, enolase is the only glycolytic enzyme in the RNA degradosome. However, two glycolytic enzymes, enolase, and PFK, are found in the *B. subtilis* degradosome. PFK is involved in one of the initial steps of glycolysis, which converts fructose-6-phosphate to fructose 1,6–bisphosphate. This step is a key regulatory step operated by the cell's energetic needs because it is activated by AMP and inhibited by ATP. Enolase converts 2-phospho-D-glycerate (2PG) to phosphoenolpyruvate (PEP), which not only produces ATPs but also transfers the energy-bearing phosphate to the sugar transport system in bacteria. The exact function of the glycolytic enzymes in the RNA degradosome is not yet known.

Recently, the intracellular localization of all proteins that are thought to be a part of the RNA degradosome has been studied (Cascante-Estepa et al., 2016). It was found that except RNase Y, which was localized to the membrane, all of other proteins were localized in the cytoplasm. This might indicate that the interaction among the RNA degradosome components is transient.

### *S. aureus* RNA degradosome

*S. aureus* is a Gram-positive coccal bacterium and a member of Firmicutes. Even though it is a part of human normal microflora found frequently in the nostril, respiratory tract, and on the skin, it can cause diverse human diseases with immense health care concern. Major infections caused by *S. aureus* range from skin infections such as abscesses and impetigo to life-threatening diseases such as pneumonia, endocarditis, toxic shock syndrome, and osteomyelitis. It is also a major nosocomial infection agent, and ~500,000 patients in hospitals contract this organism each year in the United States.

The interactions among the RNA degradosome components in *S. aureus* were studied through the bacterial two-hybrid analysis that is performed in *E. coli* (Roux et al., 2011). The *S. aureus* degradosome complex contains the same components as those of *B. subtilis*, such as RNase Y, RNase J1 and J2, polynucleotide phosphorylase (PNPase), enolase, PFK, and a DEAD-box RNA helicase. In addition, the RNase RnpA appears to be a member of the RNA degradosome complex in both *S. aureus* and *B. subtilis* (Roux et al., 2011). The interactions between these components showed conservation between the two organisms with some differences. In the *S. aureus* RNA degradosome, strong interactions appear between RNase Y and enolase, PFK and CshA, CshA and RnpA, CshA and Enolase, and RNase J1 and RNase J2. Thus, the DEAD-box RNA helicase CshA appears to play a crucial role in the *S. aureus* degradosome formation.

Khemici *et al*. examined the cleavage sequence by *S. aureus* RNase Y using the novel method EMOTE (Exact Mapping Of Transcriptome Ends; Khemici et al., 2015). The EMOTE assay is a mapping technique using high throughput sequencing to identify 5′ monophosphorylated end created by the action of endoribonucleases. The first step of this assay is a ligation of a synthetic RNA oligo to the 5′-ends of total RNA. Then, cDNA is synthesized using the sequence of the RNA synthetic oligo and sequenced through a high-throughput sequencing. This assay revealed that the preferred cleavage site by RNase Y is the 3′ side of a guanosine (58% of total cleaved sites) in adenosine and uridine-rich regions (Khemici et al., 2015). Both requirements of G and the A/U-rich region are not so strict, thus sites immediately next to other nucleotides can be cleaved (A, 30%; U, 10%; and C, 2%). Since the genome of *S. aureus* is AT rich (A+T = 67%), there can be many AU rich regions in transcripts. These results imply that other factors such as RNA structures or RNA-binding proteins might influence RNase Y to recognize its substrates.

*S. aureus* RNase Y is not essential for growth and the mutation of its gene *rny* stabilizes only some mRNAs (Marincola et al., 2012). Also, the growth of both the RNase Y deletion strain and the strain with the mutation in the active site in the HD-domain are similar to that of the wild-type strain (Khemici et al., 2015). These results suggest that the role of RNase Y in *S. aureus* might be not for the degradation of bulk RNA. The location of RNase Y appears important for its role because the strain with the transmembrane domain-deleted RNase Y grows markedly slower, implying that the degradosome formation in the cytosol is deleterious for the growth of *S. aureus* (Khemici et al., 2015).

In *S. aureus*, both RNase J1 and J2 are not essential for viability but deletion of one or both genes confers high fitness cost for growth, especially under the condition of nutritional or temperature stress (Linder et al., 2014). Interestingly, an active site mutation in RNase J1 displays the same growth defect as an RNase J1 deletion mutation, but an active site mutation in RNase J2 exhibits no growth defect. Based on this result, RNase J2 has been proposed to be a stabilizer or modulator of RNase J1 (Linder et al., 2014).

### S. *pyogenes* RNA degradosome

Another Gram-positive pathogen whose RNA degradosome components have been studied is *S. pyogenes*. *S. pyogenes* is a human pathogen that causes diverse diseases from superficial infections such as pharyngitis and impetigo to life-threatening systemic and invasive diseases such as rheumatic heart disease, toxic shock syndrome and necrotizing fasciitis (Cunningham, 2000). Almost 20% of the population carries this pathogen in their throat asymptomatically.

Before the existence of RNase Y was known, other RNase components in the degradosome such as PNPase, RNase J1, and RNase J2 were studied to examine the stability of virulence gene transcripts in *S. pyogenes* (Barnett et al., 2007; Bugrysheva and Scott, 2010). *S. pyogenes* produces many virulence factors and the regulation of these virulence factors are strictly controlled according to cellular or environmental signals. One of the signals that determine virulence gene expression *in vitro* is the growth phase. When the stability of transcripts of a M3 serotype strain, MGAS315, was examined, most virulence gene transcripts were rapidly degraded at the stationary phase. However, the stability of some transcripts of virulence genes such as *sagA, sda*, and *arcT* are greatly enhanced at the stationary phase (Barnett et al., 2007). These genes encode the precursor of the hemolysin streptolysin S, a DNase or Streptodornase, and a peptidase, respectively. In a PnpA null mutant, the transcripts of *sagA, sda*, and *arcT* are more stabilized at the late exponential phase. The transcripts of *sagA, sda*, and *arcT* show biphasic decay: slow decay at the initial period, then rapid decay after that. The *pnpA* mutation abolished the second phase of rapid decay, indicating that PNPase is responsible for the second decay at the late exponential phase.

In *B. subtilis*, RNase J1 and RNase J2 are paralogous and form a heterotetramer *in vivo* (Mathy et al., 2010). In *S. pyogenes*, both RNase J1 and RNase J2 are essential for growth, suggesting that, at least partially, their roles are not redundant and their substrates could be different *in vivo* (Bugrysheva and Scott, 2010). When RNase J1 and J2 are minimally expressed to support the growth of *S. pyogenes*, the half-lives of transcripts showing rapid decay at both exponential and stationary phase growth increase. However, in the case of the transcripts showing biphasic decay at the exponential phase and increased stability at the stationary phase, the period of the first phase of slow decay increases, but the second phase of rapid decay still maintains, unlike the *pnpA* mutation. This result indicates that PNPase and RNase J1/J2 work cooperatively and the action of RNase J1/J2 precedes that of PNPase in RNA decay.

The average half-life of *S. pyogenes* mRNAs is 1.26 min, so the turnover rate is very high even for a bacterium (Chen et al., 2013). In an RNase Y null mutant, mRNA stability increases two-fold and this indicates that RNase Y plays a major role in the global decay of mRNA in *S. pyogenes* (Chen et al., 2013). *S. pyogenes* RNase Y is also involved in the processing of certain mRNA such as the transcripts of *speB*, a cysteine protease secreted by *S. pyogenes*, and its positive regulator RopB. (Chen et al., 2012, 2013).

The structure of *S. pyogenes* degradosome and interactions among components have not yet been studied, except for the interaction between RNase Y and enolase (Kang et al., 2010).

## Mechanism of RNA decay by the gram-positive RNA degradosome

RNA can be degraded by the Gram-positive RNA degradosome in three ways. (I) Pathway initiated with RppH. RNase Y is an endoribonuclease whose activity is promoted by 5′ monophosphorylated RNA (Shahbabian et al., 2009). Hence, to be a substrate of RNase Y, 5′ triphosphate in RNA should be converted to 5′ monophosphate. The enzyme possessing this activity is the pyrophosphohydrolase RppH (Celesnik et al., 2007; Deana et al., 2008). After the action of RppH, the monophosphorylated RNA can be cleaved by the endoribonuclease activity of RNase Y, which generates a 5′-monophosphorylated end and an unprotected 3′ end (Figure 2). RNase J1 and PNPase bind to these new entry sites and degrade RNA further using their 5′–3′ exoribonuclease activity and 3′–5′ exoribonuclease activity, respectively. (II) Pathway initiated with RNase Y directly. Some RNA transcripts bypass the recognition of the 5′ monophosphorylated end by RNase Y and are cleaved regardless of the 5′ phosphorylated status. That is, RNase Y has 5′ end independent endoribonuclease activity against those transcripts (Yao et al., 2011). RNase E also has this 5′ end independent endoribonuclease activity and it was speculated that this 5′ end bypass might be caused by certain tertiary folds of RNA substrates (Schuck et al., 2009; Kime et al., 2010). (III) Pathway initiated by RNase J1. RNase J1 could start RNA decay with its 5′ independent endoribonuclease activity. Then, the exoribonuclease activities of RNase J1 and PNPase degrade the cleaved RNA further (Yao and Bechhofer, 2010; Yao et al., 2011).

## Role of RNase Y in the virulence of gram-positive pathogens

RNase Y plays a crucial role in the virulence of the important Gram-positive pathogens *S. aureus* and *S. pyogenes* (Kaito et al., 2005; Kang et al., 2010). Since RNase Y orthologs do not exist in eukaryotic cells, RNase Y can be a target for the development of new antimicrobial agents.

In *S. aureus*, RNase Y was studied first as the name of CvfA. Kaito *et al*. screened *S. aureus* mutants to discover genes affecting the virulence using a silkworm model. The RNase Y ortholog was one of the discovered genes and named conserved virulence factor A (CvfA; Kaito et al., 2005). The virulence of the CvfA null mutant was also attenuated in a murine intraperitoneal infection model. Later, the same group demonstrated that the phosphodiesterase activity of CvfA is necessary for the virulence of *S. aureus* (Nagata et al., 2008).

In an RNase Y null mutant, the expression of several virulence genes such as *lukD, hlgC, nwmn0218* were lower than that in the wild type. The expression of these virulence genes is controlled by the *saePQRS* operon, but RNase Y influences the expression of the virulence genes independently of the *saePQRS* operon (Marincola et al., 2012). The *spa* transcript, which encodes Protein A, was expressed more than 8-fold lower in the RNase Y null mutant compared to the wild-type and this is due to the stabilization of the *agr* quorum-sensing system in the mutant strain (Khemici et al., 2015). The *spa* gene is negatively regulated by the *agr* quorum-sensing system (Fechter et al., 2014).

The virulence of the RNase Y null mutant of *S. pyogenes* is also highly attenuated in a murine subcutaneous model, suggesting that some virulence gene transcripts might be degraded or processed by RNase Y. Indeed, RNase Y influences the transcript abundance of virulence genes depending on growth phases and nutritional status (Kang et al., 2010). The differential expression of virulence genes in the RNase Y mutant over the wild-type was most profound in the peptide-rich, sugar-poor C medium at the stationary phase. In this condition, the transcripts of the genes of streptokinase, the CAMP factor, streptolysin O, and M protein are more abundant, but those of SpeB, a mitogenic factor, and streptolysin S are less abundant in the RNase Y null mutant than those in the wild-type HSC5, an M14 serotype strain. This result is interesting because the transcripts of the genes of mitogenic factor (aka DNase or Streptodornase) and streptolysin S (SagA) are stable at the stationary phase in the MGAS315 strain (Barnett et al., 2007), and the SpeB production of MGAS315 is much weaker than that of HSC5.

## Discussion and perspective

Membrane association seems to be a common feature of the degradosome in bacteria (Górna et al., 2012). This occurs through the scaffold proteins RNase E in *E. coli* and RNase Y in *B. subtilis*. In fluorescence microscopy studies, the *E. coli* degradosome complex appear to form a filamentous structure that is helical along the interior surface of the membrane (Taghbalout and Rothfield, 2007, 2008). The region responsible for membrane binding is a small segment in the C-terminal half of RNase E forming an amphipathic helix (Khemici et al., 2008). This segment alone can bind *E. coli* lipid vesicles by forming a stable amphipathic alpha-helix. When this amphipathic helix formation is disrupted by amino acids substitution, the mutated RNase E cannot bind the membrane. This causes slower growth, indicating that the membrane association of RNase E is important for its function or RNase E dissociated from the membrane is toxic to *E. coli*. The dissociation of RNase Y from the membrane also slows bacterial growth down (Khemici et al., 2015).

The RNA degradosome localization indicates that the RNA substrates of the RNA degradosomes are near the membrane. It has been believed that transcription and translation are coupled in bacteria, so they occur near the chromosome. However, a large portion of cellular transcripts is the substrates of the degradosome, so the RNA substrates should be located near the membrane. Hence, some bacterial RNA might not be translated at the same time of transcription; some transcripts might be detached from the chromosome and diffuse to the cytoplasm or to the membrane to be translated (Nevo-Dinur et al., 2011). Otherwise, mRNA might diffuse to the cytosol and reach to the membrane if the translation cannot occur due to mRNA damage or translation inhibition.

Deletion of RNase Y in *B. subtilis* and *S. pyogenes* increases their average RNA half-life 2-fold, indicating that RNase Y in these Firmicutes is responsible for bulk RNA degradation (Shahbabian et al., 2009; Chen et al., 2013). However, the mutation of *S. aureus* RNase Y stabilizes only some mRNAs (Marincola et al., 2012). Therefore, all RNase Y in Firmicutes might not be the major endoribonuclease involved in bulk RNA decay. Unlike RNase E, RNase Y is not essential for growth. Other new ribonucleases known to exist in the Gram-positive RNA degradosome are RNase J1 and J2. However, the presence of RNase J1 and J2 in the Gram-positive degradosome is a matter of some controversy. While an RNase Y-RNase J1 interaction and an RNase P-RNase J1 interaction were shown by Commichau et al. (2009) and Newman et al. (2012), these interactions were not seen by Mathy et al. (2010). RNase J1 and J2 are not essential for growth in *B. subtilis* and *S. aureus*, but both are essential in *S. pyogenes* [Bugrysheva and Scott, 2010; Figaro et al., 2013; Linder et al., 2014].

RNase Y in Gram-positive bacteria is not essential for growth, but RNase Y null mutants grow slower. It has not been studied whether the phenotype is caused by the endoribonuclease activity or the degradosome formation activity. The coiled-coil domain at the N-terminal side of RNase Y seems involved in the interaction with other degradosome components, so detailed structural and functional studies of the coiled-coil region might confer the possibility to separate the degradosome formation activity from the endoribonuclease activity of RNase Y.

The Gram-negative RNA degradosome contains one glycolytic enzyme enolase, but the Gram-positive degradosome possesses two glycolytic enzymes, enolase and PFK. The exact function of these glycolytic enzymes in the RNA degradosome has not yet been revealed. If the role of glycolytic enzymes is simply to control RNA decaying activity based on cellular energy status, they could be replaced by simpler signaling molecules regulating protein activity allosterically such as ATP, ppGpp, c-di-AMP, and c-di-GMP. This type of regulation seems to be used already in the *E. coli* degradosome. The activity of PNPase is allosterically regulated by ATP *in vitro* (Del Favero et al., 2008). Thus, the role of glycolytic enzymes in the degradosome might be more sophisticated than just controlling RNA metabolism by a cellular energy level. There might be communication between the RNA degrading pathway and major energy-producing pathways through the glycolytic enzymes. In *E. coli*, enolase in the degradosome influences the activity of energy-producing metabolic pathways. Removal of enolase from the *E. coli* degradosome by deleting the enolase-binding site in the RNase E C-terminal half influences the turnover of many mRNAs, specifically those encoding enzymes involved in sugar metabolism (Bernstein et al., 2004), and the *ptsG* mRNA that encodes the glucose transporter (Morita et al., 2004). The glucose transporter system uses the product of enolase, PEP to generate phosphoglucose. The disruption of interaction between enolase and RNase E also influences growth rates in minimal media containing different carbon sources. In *B. subtilis*, RNase Y performs an endonucleolytic processing of the *gapA* operon transcripts (Commichau et al., 2009). In *S. pyogenes*, the transcript abundance of glycolytic enzymes such as glucose-6-phosphate isomerase, PFK, enolase, pyruvate kinase, and L-lactate dehydrogenase increases more than two-fold in the RNase Y null mutant compared to that of the wild-type (Kang et al., 2010). The glycolysis is the major energy-producing pathway in the anaerobe *S. pyogenes*. In addition, *S. pyogenes* RNase Y differentially responds to two different nutritional elements, carbohydrates and proteins. Normally, RNase Y null mutants produce more streptokinase, the CAMP factor, and M protein, and less SpeB at the stationary phase in the sugar-poor, protein-rich C medium. However, when more sugar is added to the medium, the mutant produces the wild-type level of streptokinase, the CAMP factor, and M protein. When more proteins are provided, the mutant produces the wild-type level of SpeB. This nutritional source-dependent virulence gene expression may be due to the two glycolytic enzymes in the *S. pyogenes* RNA degradosome.

Since the Gram-positive degradosome was only recently discovered, much study is necessary to uncover the role of the degradosome in RNA metabolism, in the regulation of gene expression, and in the virulence of Gram-positive pathogenic bacteria. Also, more study should be done to understand the structure and function of the degradosome. Questions below could be asked for future studies on the Gram-positive degradosome. Does the Gram-positive degradosome form a helical filamentous structure like the *E. coli* degradosome? Which parts of RNase Y are used to interact with each component? What is the stoichiometry among the components? What is the role of the membrane localization of the degradosome? Why do two similar enzymes RNase J1 and RNase J2 exist and what are their roles in the degradosome? Why do two glycolytic enzymes exist in the degradosome and what are their roles?

## Author contributions

The author confirms being the sole contributor of this work and approved it for publication.

### Conflict of interest statement

The author declares that the research was conducted in the absence of any commercial or financial relationships that could be construed as a potential conflict of interest.
